# Designing and building OSCEBot ® for virtual OSCE – Performance evaluation

**DOI:** 10.1080/10872981.2023.2228550

**Published:** 2023-06-22

**Authors:** Daniela S. M. Pereira, Filipe Falcão, Andreia Nunes, Nuno Santos, Patrício Costa, José Miguel Pêgo

**Affiliations:** a Life and Health Sciences Research Institute (ICVS), School of Medicine, University of Minho, Braga, Portugal; b ICVS/3B’s, PT Government Associate Laboratory, Braga, Portugal; c iCognitus4all – IT Solutions, Braga, Portugal

**Keywords:** Chatbots, Medical education, Natural language processing, Technology-enhanced learning, BERT, OSCEs

## Abstract

With AI’s advancing technology and chatbots becoming more intertwined in our daily lives, pedagogical challenges are occurring. While chatbots can be used in various disciplines, they play a particularly significant role in medical education. We present the development process of OSCEBot ®, a chatbot to train medical students in the clinical interview approach. The SentenceTransformers, or SBERT, framework was used to develop this chatbot. To enable semantic search for various phrases, SBERT uses siamese and triplet networks to build sentence embeddings for each sentence that can then be compared using a cosine-similarity. Three clinical cases were developed using symptoms that followed the SOCRATES approach. The optimal cutoffs were determined, and each case’s performance metrics were calculated. Each question was divided into different categories based on their content. Regarding the performance between cases, case 3 presented higher average confidence values, explained by the continuous improvement of the cases following the feedback acquired after the sessions with the students. When evaluating performance between categories, it was found that the mean confidence values were highest for previous medical history. It is anticipated that the results can be improved upon since this study was conducted early in the chatbot deployment process. More clinical scenarios must be created to broaden the options available to students.

## Background

Chatbots – ‘Can Machines Think like Doctors do?’

Chatbots, also known as artificial conversation entities, interactive agents, smart bots, and digital assistants are systems that can communicate with users by using methods and algorithms from two Artificial Intelligence (AI) domains: Natural Language Processing (NLP) and Machine Learning (ML) [1–3].Until recently, the most notable instance was the usage of chatbots as personal assistants, like Google Assistant and Apple’s Siri [1,3]. While chatbots may be employed in various fields, they are particularly relevant in the digital transformation of education since they provide both students and teachers with new resources [4–6]. Considering changes in the educational technology landscape, interactions with students must now be more individualized to support their unique learning styles and meet their various needs. The challenge of meeting the requirements of this generation is one that educators are currently facing. Chatbots might be the solution to engaging younger generations naturally and natively [7,8]. Recent studies have shown that virtual conversational agents act as study partners or instructors, and can improve students’ academic performance 9, 10]. Higher Education (HE) learning environments have changed significantly over the past few decades due to the use of Technology-Enhanced Learning (TEL) systems, where cutting-edge technologies and artificial intelligence are used to allow for greater flexibility, personalization, engagement, and motivation of learners [11]. The future of education could be transformed by educational chatbots (ECs). With the help of a virtual assistant that simulates human interactions, these chatbots are designed to offer personalized learning [3]. OpenAI’s ChatGPT^TM^ (Chat generative pre-trained transformer), which was introduced in November 2022, is one illustration of this. Since ChatGPT^TM^is tailored to each learner’s needs and preferences, it may support learners’ autonomy and enhance their learning experiences. This chatbot is particularly crucial for self-learning. However, numerous people have brought attention to moral concerns like plagiarism, cheating, and data security. Additionally, there have been some reports regarding ChatGPT^TM^ not giving bibliographical references [12].

## Conceptual framework

### Chatbots in medical education

The practice of medicine is transforming and evolving at a remarkable rate. Due to the multifaceted demands of an aging population, the variety of available treatment choices, the multidisciplinary character of care, among others, today’s healthcare systems are considerably more complex than they were 20 years ago [13]. As a result, medical education has also been adjusted to train doctors capable of dealing with this and other changes. Several factors, such as the evolving healthcare landscape, the shifting nature of the physician’s position, altered societal expectations, the fast advancement of medical knowledge, and the variety of pedagogical approaches, impact how medical education is delivered [14]. The use of technology in medical education serves a variety of educational purposes, including facilitating the acquisition of fundamental knowledge, communication skills, enhancing decision-making, increasing perceptual variety, improving skill coordination, practicing for uncommon or important events, learning teamwork, and enhancing psychomotor abilities [14–16]. In medical education, conversational agents are mostly used as virtual patients (VPs) that simulate clinical scenarios and enable training in interviewing and communication techniques.This type of system has seen a surge in popularity over the past 10 years, largely due to the particular benefits associated with it, including its ability to provide personalized exercises in a safe, non-threatening environment and performance assessment with opportunities for reflection and feedback [15]. During the pandemic, the medical education curriculum went fully digital, undermining the attainment of these important skills for students to be successful in their academic careers. One of these skills is the ability to interview the patient, obtaining the patient’s clinical information effectively while maintaining a trusting relationship with the patient – the so-called history taking.

## Why are history taking skills so important?

Clinical competencies, such as clinical reasoning, history-taking skills, diagnosis, physical examinations, and communication/professionalism skills, are crucial in medical education curricula. These skills allow students to manage knowledge to solve clinical problems while enabling them to establish a trusting relationship [17]. Given that conducting medical interviews is the clinical professional’s most common activity, history taking is crucial for medicine practice [18]. The importance of clinical history in arriving at a good diagnosis is crucial, and the importance of reaching a correct diagnosis cannot be overstated [19,20]. During their career, clinicians will interview between 100,000 and 200,000 patients [18]. A noteworthy example of this is that around 60 to 80% of the information that is relevant for a diagnosis is gathered in medical history and 76–90% of all diagnoses [18,19] are determined solely based on the information that physicians acquire from the clinical interview. Hence, any skilled physician must possess the core abilities to gather a patient’s medical history and apply that knowledge to make accurate diagnoses [21]. History taking is one of the earliest fundamental skills taught in medical school. This skill requires a systematic methodology and a clearly defined structure, which is vital for the clinical interview. In addition to following this structure, the student should also pay close attention to nonverbal cues, such as eye contact, silence, posture, etc. Appendix 1 describes this methodology for a patient presenting leg pain. This set of skills is usually trained in laboratory conditions or in the wards and assessed using Objective Structured Clinical Examination (OSCE) [22] type of exams. In these assessments, students interact with a simulated patient (SP), a person trained to play a patient’s role according to a script. Despite being considered a high validity and reliability form of assessment, OSCE usually demand significant financial and human resources and infrastructure availability and time for planning and logistics [17]. Additionally, training SP and deploying OSCE can be expensive, as one exam costs may amount to Eur 10 000 [23] and close to Eur 100 per examinee [24]. Furthermore, students have fewer opportunities to train and prepare for this kind of assessment and often have brief contact with SPs prior to the actual exam. This can negatively affect the evaluation and learning process since it can be inadequate to develop critical skills for medical practice, such as communication skills and empathy, that can influence patients’ satisfaction and improve health outcomes [23,25]. The development and subsequent evaluation of a chatbot to aid in and assess the growth of clinical skills in medical students will be discussed in this paper.

## Materials and methods

### Concept- project requirements

The major goal of this project is to create a fully functional conversational agent prototype to assist medical students in improving their clinical skills, specifically the clinical interview, and, subsequently, allow to cover a training opportunity gap for OSCEs. The educational objectives of the present system were defined in accordance with those specified for the OSCEs:

1. Become familiar with the work environment present in everyday clinical practice;
2. Develop clinical problem-solving skills,
3. Develop and enhance clinical interviewing skills;
4. Develop clinical information summary skills;
5. Increase diagnostic interpretation skills;
6. Improve confidence in decision-making and self-efficacy;
7. Identification of appropriate therapeutic interventions.

To achieve these goals, the project’s educational strategy used a student-centered, case-based learning technique, which required the students to take ownership of building their understanding of the clinical settings. This approach is founded on the notion that learning is maintained and remembered longer when done in a context that represents realistic workplace circumstances to encourage students to apply knowledge obtained from the classroom or through additional research to solve the case [26].

Regarding the development of the current educational chatbot, several guiding concepts were selected as fundamental for the successful application of this system:

1. The original design and development of the chatbot should be built through co-creation, bringing together instructors, developers and end-users to create a chatbot that fits the demands and needs to help student’s preparation for OSCEs.
2. The framework should offer on-site data storage and all the tools required for continuing model training.
3. The system needs to be simple for non-technical individuals to employ. It should also be possible to customize cases and patients without scripting or programming knowledge and to increase the number of cases without sacrificing user experience standards.
4. The chatbot must recognize user input in natural language, respond accordingly and determine the user’s intent to offer a response appropriately using, following the NLP principles, human-like language (output).

Like Kumar [27], the Reliability, Interpersonal communication, Pedagogy, and Experience (RIPE) principles were used to identify the attributes considered essential for chatbot design (Table 1).

**Table 1.** RIPE Principles. Adapted from Kumar, 2021.

	DESCRIPTION	CONTEXT
Reliability	The chatbot should be simple to use and accessible through a secure platform so learners can rely on it to get ongoing feedback securely.	Using specific software for student assessment (quizOne) improves the interaction between the student and the software while maintaining information security.
interpersonal communication	The chatbot (hence, patient) should establish a relationship with the learner using NLP, figures of speech, popular expressions, etc.	To emulate a real patient, chatbots must mimic interpersonal communication.
pedagogic	The chatbot provides learning content and activities that align with the learning. Therefore, while facilitating a personalized learning platform, the chatbot should also embody active learning and communication strategies that allow the instructor to monitorlearning progress learning progress	After the interaction between the chatbot and the student, it can be viewed by teachers and educators. In addition, at the end of the interaction, students will be able to review the clinical interview and put the most likely diagnosis hypotheses. After that, the chatbot will provide immediate feedback and identify the strengths and weaknesses of the interview.
experience	The chatbot should be used on a preferred communication platform and mimic how patients naturally communicate. To increase emotional engagement, affective contact such as greetings, comedy, emoticons, and/or empathy should be used.	The chatbot will use NLP to better engage students. Students will also be presented with opportunities for empathetic interaction, where the patient presents their concerns, and the student is expected to respond appropriately.

### Building the virtual patients

For the sake of comparability and efficiency of the chatbot training, we focused on symptoms that followed the SOCRATES approach. For this project, several Virtual Patients (VPs) were created covering a broad range of key symptoms: chest pain, lumbar pain, leg pain and dyspnea. Scripts were developed by clinical educators experienced in developing SP clinical cases scripts for use in OSCE and reviewed by a senior clinical educator. Cases were presented randomly to students to avoid bias regarding the case’s complexity or students’ confidence in any given subject.

### Chatbot architecture

This chatbot is a web-based program that responds to queries from medical students. The chatbot answers the user by extending a friendly greeting before responding appropriately to the query posed, such as ‘what brought you here?’ In this instance, the answer would be the primary complaint (Appendix 2). The user should continue asking the chatbot questions about his symptoms, family history, personal history, and other topics, much like in a clinical interview. The chatbot will find the most appropriate trigger phrase or node and respond using a flexible pre-written script that medical specialists have approved. The prewritten script was based on the SOCRATES approach. While formulating questions to verify the exploration of the chief complaint (duration, intensity, location); clinical, social, and family history. Regardless of whether the SP suffers from leg or chest pain, the question regarding intensity, for example, will be the same. The script was built for one case, and each time a new case was introduced an experienced medical educator checked if the information was correct and if the information inserted in the script would be enough for the student to reach a diagnostic hypothesis. The chatbot will prompt the learner to reword their query if it cannot find an appropriate response or understand the question.

**Figure 1.**
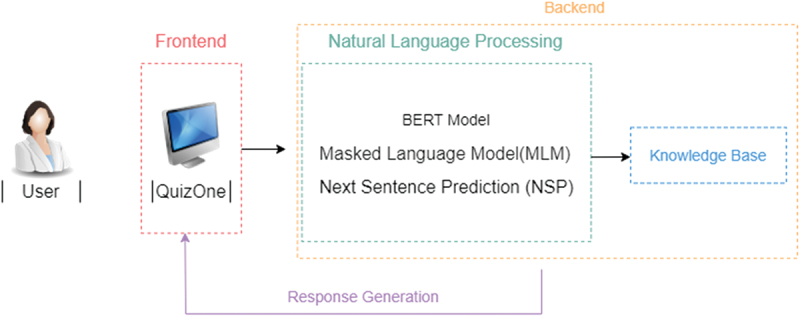
Chatbot architecture.

The system’s architecture of the chatbot system is shown in Figure 1

The architecture of the developed chatbot is composed of:

- Frontend
- Backend
- Knowledge Base Module
- Conversational Module

The first module, which offers a user-friendly interface, represents the presentation layer (frontend) created in this particular scenario using the Angular framework [28]. Operations that are not visible to the end user are managed on the backend. The frontend communicates with the backend through an API gateway, and this gateway communicates with microservices, one of which is the Python service that uses SBERT. Due to scalability, maintenance of the analysis algorithms, speed of execution, and better match customer demand, this module operates in the background; it manages business logic and data storage while collaborating with the knowledge base. In this case, the backend was developed using.NET. The Knowledge Base Module is a unique database used for managing knowledge and information, where a server processes data. Regarding the Conversational module, based on a focused Internet search, we evaluated potential NLP tools and software solutions which could support the construction of a virtual patient. The chatbot was created with the ability to handle a diverse range of cases and randomly select one when deployed. The SentenceTransformers, or SBERT, framework was used to develop this chatbot. This is a Python framework for state-of-the-art embedding of text, phrases, and images [29]. This framework can be used to create text embeddings and phrases in various languages. The pretrained BERT(short for ‘Bidirectional Encoder Representations from Transformers’) network created by [30-] the basis of the SBERT model – enables bidirectional training to be applied to a word sequence using a transformer. BERT has already been investigated, outperforming other frameworks [31]. It works by encoding a sequence of words (e.g., a sentence) into a fixed-length vector representation, which can then be used as input to a downstream task such as text classification or question answering. By considering the words that come before and after them and how they relate to the remainder of the sentence, it is intended to comprehend the context and meaning of the words in a particular sentence. To do this, BERT uses an attention mechanism to weigh the importance of different words in the input based on their relevance to the task. For example, in a question answering task, the model might pay more attention to the words in the question when generating an answer. These embeddings can then be compared, for instance using cosine similarity, to find sentences having a common meaning. This is helpful for semantic textual similarity searches, semantic search, and paraphrase mining. It is also open source and might be usable for real-time evaluation via an API. The preexisting models can also be modified to fit other scenarios, making this tool highly adaptable. To enable semantic search for various phrases, SBERT uses siamese and triplet networks to build sentence embeddings for each sentence that can then be compared using a cosine-similarity [29]. This paradigm is especially appealing to the current study since students can ask questions in various ways, meaning different inquiries might have a semantically similar meaning. In this work, SBERT was also used as a classifier. Through the content and context similarity between the questions asked by the students and the questions present in the script, the model classifies the questions according to the pre-defined blocks (main complaint, explore the main complaint, previous clinical history, social history, family history, interview closing). By setting the level of similarity between the question asked and the question present in the script, the model applies a decision tree based on the confidence value (CV). Below a certain value, the bot will produce a response asking to rephrase the question, to avoid misplaced answers.

### Sample

The study sample comprised the log file of 13 medical students from the last four years (clinical training) of the medicine program at the School of Medicine of the University of Minho, of whom 11 were females (73.3%). This sample includes log files for 13 chatbot sessions. The average self-reported English level of students was B2, according to the European framework, ranging from A1 to C2. One user was omitted from the sample due to not being comfortable with producing a discussion with the chatbot using the English language. The 813 interactions between the students and the bot were then analyzed, of which 27 interactions for the remaining 12 students were eliminated (due to using Portuguese, incomplete sentences, etc.).

#### Data analysis

The 813 interactions were reviewed by two independent judges, who assessed whether the bot had correctly identified the question and provided the correct answer based on its script for clinical cases. If the bot identified the correct question, it was expected to give the correct answer. Cohen’s κ was run to determine the agreement level between the two independent judges.

The confidence value (CV) for each interaction was calculated using SBERT, a machine learning model that employs siamese and triplet networks to generate sentence embeddings for each sentence [29]. These embeddings can then be compared using cosine similarity to determine the similarity between the student’s questions and the questions present in the clinical case’s script. The database was created, and performance metrics were calculated using Microsoft Excel. IBM SPSS version 27 and MedCalc statistical software were used to compute the ROC curves, determine the optimal cutoff values, and compare cases and categories within cases.

ROC curves were computed to understand the optimal cutoff point for our model [32]. The Youden index and the closest top-left distance were used as metrics to determine the optimal cutoff value. Once identified, the optimal cutoff point was used to compute various performance metrics, including precision, recall (sensitivity), accuracy, specificity, and F1 score. The performance metric values were determined considering the CV, as generated by the framework. The CV serves as an indication of the level of confidence in the bot’s responses. The optimal cutoff point was used to establish the CV for each case or category.

There are four possible results when a classifier, classification model, and an instance are provided: True Positives, True Negatives, False Positives, and False Negatives. True Positives were correct answers provided by the bot where the confidence value was above the cutoff value. True Negatives were determined when the bot could not answer the question and the confidence value was below the cutoff value. False Positives were defined as instances when the bot incorrectly identified the question, and the confidence value was above the cutoff value. Finally, False Negatives were situations where the bot should have provided an answer because the question was present in the script, but it did not. The confidence value was below the cutoff value.

A two-by-two confusion matrix, also known as a contingency table, can be built to express the dispositions of a set of cases given a classifier and a set of examples (the test set). Many standard performance metrics, such as precision, recall(sensitivity), accuracy, specificity, and F1 score, are built from this matrix. These metrics allow the evaluation of the model’s overall performance and understanding of how well it could identify positive cases [32]. Table 2 shows a confusion matrix.

**Table 2.** Confusion Matrix.

		True Class	
Hypothesized class		**+**	-
	**+**	True Positives	False Positives
	-	False Negatives	True Negatives

The performance measures were calculated with the assistance of the aforementioned table and are presented in Table 3.

**Table 3.** Performance Measures [33,34].

Measures	Meaning	Equation
Accuracy	Efficiency of an algorithm to accurately categorize examples from a given dataset.	$Accuracy=\frac{TP+TN}{\left(TP+TN+FP+FN\right)}\left(1\right)$
Precision	Performance between the information asked and the response provided.	$Precision=\frac{TP}{\left(TP+FP\right)}\left(2\right)$
Recall/Sensitivity	How well a system performs while recovering data.	$Recall=\frac{TP}{\left(TP+FN\right)}\left(3\right)$
F Score	Evaluation of a model’s performance by considering both precision and recall.	$FScore=\frac{\left(2\left(Precision\times Recall\right)\right)}{\left(Precision+Recall\right)}\left(4\right)$
Specificity	Ability of the system to identify true negatives.	$Specificity=\frac{TN}{\left(TN+FP\right)}\left(5\right)$

The differences between clinical cases and question categories were also investigated, as well as the performance metrics of the bot in the different cases and categories, this was achieved by ANOVA and ROC (receiver operating characteristic) curve computation.

## Results

### Dataset

Cohen’s κ was run to determine the agreement level between two independent judges on whether the bot had correctly identified the question and, consequently, given the correct answer. There was substantial to almost perfect agreement between the two judges, κ = .804 (95% CI, 0.763 to 0.845), *p* < .001[35].

### Determining the optimal cutoff value

The performance of the bot was evaluated using a ROC curve. The ROC curve plots the true positive rate (sensitivity) on the y-axis and the false positive rate (1 - specificity) on the x-axis for all possible classification thresholds [36]. The area under the curve (AUC) was 0.864, indicating that the Chatbot could accurately identify the appropriate questions and provide accurate answers [37].

To determine the optimal cutoff value for our model, we used two methods: the Youden index [38] and the closest top-left distance. Overall, the two methods produced very similar results, with an optimal cutoff value of approximately 0.86 in both cases (0.8597 for the Youden Index and 0.8577 for the closest top-left distance). The precision, recall, and F1 score was highest at this cutoff value, with values of 0.85, 0.663, and 0.72, respectively. There were slight differences in the results of the two methods (Table 4).

**Table 4.** Performance measures for Youden Index and Closest top-left distance cutoff values.

	Youden Index	Closest top-left distance
Cutoff Value	0.8597	.8577
Precision	0.854	.853
Recall	0.633	.635
Accuracy	0.418	.419
F1 Score	0.727	.728
Specificity	0.790	.786
Sensitivity	0.633	.635

The closest top-left distance produced slightly higher sensitivity and accuracy values than the Youden index. However, these differences were relatively small and are not expected to significantly affect the model’s overall performance.

### Performance between cases

The performance of the bot was evaluated using three clinical cases. The case concerning chest pain was defined as case 1, lower limb pain as case 2, and dyspnea refers to case 3. The descriptive statistics for the cases are presented in Table 5.

**Table 5.** Descriptive Statistics for cases.

		N	%
Clinical Case	1	186	22.9
	2	357	43.9
	3	270	33.2
	Total	813	100

The mean CV for clinical case 1 was 0.84 (SD = 0.11). For clinical case 2, it was 0.86 (SD = 0.11), and for clinical case 3, it was 0.87 (SD = 0.97).

**Figure 2.**
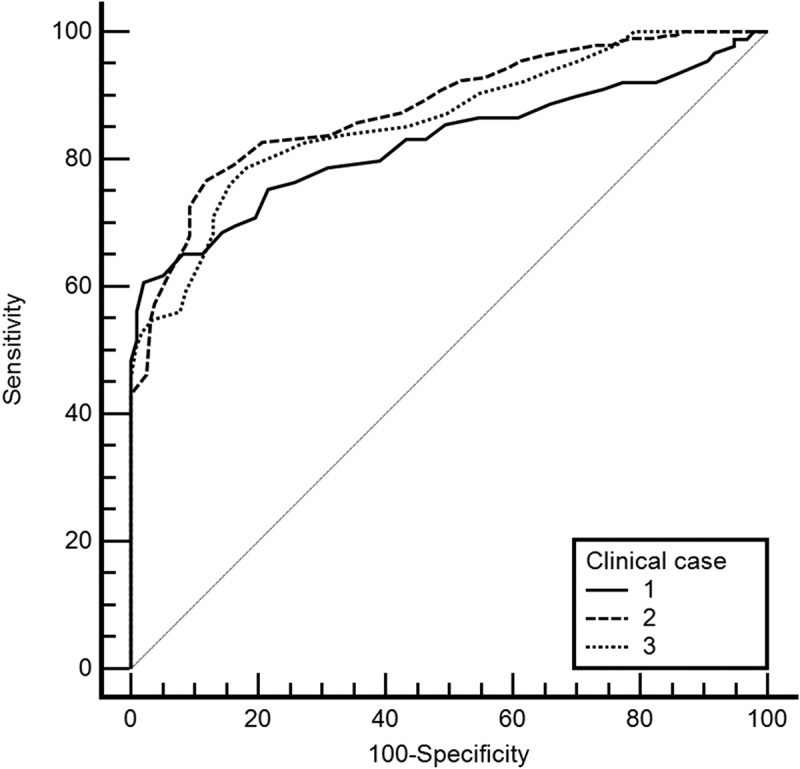
ROC curves for each case.

As expected, the mean CV value increases as the clinical cases progress because feedback from the first case was used to improve and add to the scripts of the subsequent cases. As a result, the mean CV value increases from case 1 to case 2 to case 3. A one-way ANOVA was performed to compare the effect of the clinical case in confidence value. The test revealed that there was a statistically significant difference in CV between at least two groups (F(2,810) = 3.28, *p* = 0.038, η_p_^2^ = 0.008). Tukey’s HSD Test for multiple comparisons found that the mean value of CV was significantly different between clinical case 1 and 3 (*p* = 0.031, 95% C.I. = [-,0498,0019]). There was no statistically significant difference between clinical case 2 and case 1 (*p* = 0.403) and clinical case 2 and case 3 (*p* = 0.268). This can also be observed by the computation of ROC Curves (Figure 2).

Table 6 shows each case’s AUC (Area under the curve) and optimal cutoff values. The performance metrics were also computed for the optimal cutoff value.

**Table 6.** AUC and performance metrics per case.

	Case 1	Case 2	Case 3
AUC	0.83	0.88	0.86
Cutoff Value	91.50	86.50	86.50
Precision	0.91	0.87	0.86
Recall	0.51	0.57	0.61
Accuracy	0.63	0.45	0.68
F1 Score	0.66	0.69	0.72
Specificity	0.89	0.83	0.81
Sensitivity	0.51	0.57	0.61

Note: performance values computed for the optimal cutoff value.

The Friedman test was performed on the performance measures (precision, recall, accuracy, F1 score and specificity) across different clinical cases. The results indicate that the performance measures were not significantly different across clinical cases, χ2(2) = 0.4, *p* = 0.819.

### Performance between categories

The interactions between the students and the bot were classified based on their type. Greeting interactions (such as ‘hello’ or ‘hi’) were placed in category 1, while interactions exploring the main concern were placed in category 2 (‘On a scale of 1 to 10, how severe is your pain?’). Questions about past medical history (‘have you ever had surgery before?’) were classified as category 3; social history (‘do you smoke?’) as category 4; family history (‘has anyone in your family ever had cancer?’) as category 5; and closing the interview (‘is there anything else you need from me?’) as category 6.

Table 7 describes the frequencies of each category in all the interactions.

**Table 7.** Frequency distribution of categories.

Category	N	%
1	17	2.1
2	495	60.9
3	114	14.0
4	143	17.6
5	35	4.3
6	9	1.1

Due to the low frequency of categories 1, 5, and 6, the analysis was focused on categories 2 (main complaint), 3 (Previous clinical history), and 4 (Social history) (*N* = 752).The mean CV for category 2 (main complaint) was 0.85 (SD = 0.10). For category 3, previous clinical history was 0.87 (SD = 0.10), and for category 4 (social history) was 0.86 (SD = 0.13).

As expected, a negative association was observed between the number of questions asked and the mean confidence value. This is likely since a larger number of questions (*N* = 495) was asked about the main complaint, leading to more errors and a corresponding decrease in the mean confidence value compared to other categories. There were no statistically significant differences between categories means as determined by one-way ANOVA (F(2,749) = 0.758, *p* = . 469, ηp2 = 0.002).

**Figure 3.**
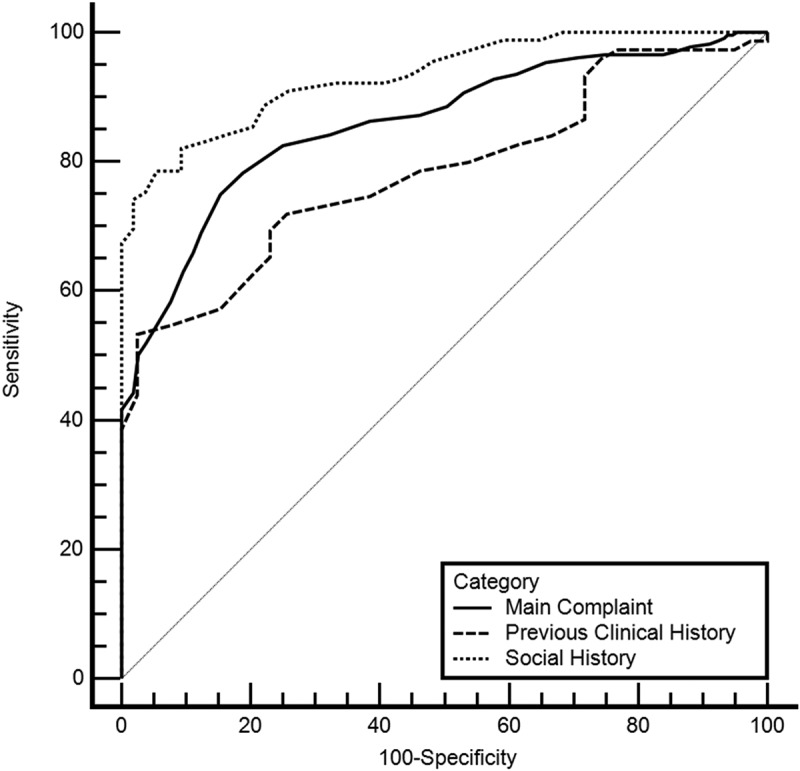
ROC curves for each category.

ROC curves were also computed for each category (Figure 3).

Table 8 shows the AUC and optimal cutoff values for each category. The performance metrics were also computed for the optimal cutoff value.

**Table 8.** AUC and performance metrics per category.

	Category 2	Category 3	Category 4
AUC	.86	.78	.93
Cutoff Value	.87	.92	.87
Precision	0.86	0.88	0.86
Recall	0.62	0.59	0.61
Accuracy	0.68	0.67	0.68
F1 Score	0.72	0.70	0.72
Specificity	0.80	0.83	0.80
Sensitivity	0.62	0.59	0.62

Note: performance values computed for the optimal cutoff value.

As can be observed by both the ROC curves and Table 8 category 4 has a higher AUC value and overall, the best performance within the categories.

The Friedman test was performed on the performance measures (precision, recall, accuracy, F1 score and specificity) across different categories. The results indicate that the performance measures were not significantly different across the different categories, χ2(2) = 0.5, *p* = 0.779.

## Discussion

The primary objective of this study was to conduct a preliminary assessment of the OSCEBot® model for use in OSCEs training. The optimal cutoffs were determined, and the performance metrics were then calculated for each case and category. ANOVA and ROC curve computation was also used to gain a deeper understanding of the model’s performance on different clinical cases and categories and identify areas where it may be necessary to improve its accuracy.

Concerning the computation of performance measures for the overall best cutoff value, compared to the Youden index, the closest top-left distance produced somewhat higher scores for sensitivity and accuracy. The model’s performance is not anticipated to be greatly impacted by these relatively minimal changes.

Regarding the performance between cases, case 3 has a higher average CV value (0.87) than the other 2 cases. The continuous improvement of the cases could explain this following the feedback acquired after the sessions with the students. Since case 1 was the first to be developed and the one from which the following cases were developed, it is expected to have the lowest average CV. This is also corroborated by the differences in the ANOVA and the performance measures that are generally better in case 3. However, when we look only at the AUC, case 2 has a higher value, larger area, and therefore better average performance [32].

When evaluating performance between categories, it was found that the mean CV was highest for category 3 (previous medical history), followed by category 4 (social history) and category 2 (main complaint). The mean CV values for these categories were 0.87, 0.86, and 0.85 respectively. While category 2 had the lowest mean CV value, it also had the highest associated sample size. This suggests that exploring the main symptom was a primary focus of the students in this study. The performance measures among the three categories were relatively similar, with categories 2 and 4 showing slightly better results than the others. Additionally, category 4 had a higher AUC value.

It is crucial, however, to emphasize that because one-dimensional performance metrics are naturally extremely variable and do not reveal the complete story, especially when they are approximated using scant data, they should only be used as fundamental suggestions [39–41].

In the present study, the performance of the OSCEBot ® model was assessed, for example, by its ability to provide accurate and relevant information to users. In addition, it was possible to highlight differences in performance regarding the case and category. This study has allowed us to make a preliminary evaluation of the bot, thus enabling the continuous improvement of the model, verifying, and correcting the weak points and identifying the modules where modifications should be applied.

Additionally, some restrictions to be noted in this study were found. Initially, sample size: the study’s use of a small sample of students and cases may have prevented the findings from being applied to a larger population. Also, because the interactions with the students only focused on the clinical interview process, they were not adequately prepared for all the context and tasks necessary for an OSCE (such as a physical examination and nonverbal communication), nor were characteristics that would occur in a real situation considered (e.g., time restrictions). One potential limitation is using a language other than the students’ native language. Despite most students expressing proficiency in English, there is the possibility for grammatical and orthographic errors that could potentially compromise the results presented by the framework.

## Conclusion

Although some chatbots for training OSCES already exist, the present work focused on developing it together with key players. In addition, the system is integrated into a closed-used platform already consolidated in medical education, maintaining data security and confidentiality. To the best of the author’s knowledge, no preliminary study of this kind has ever been conducted for a chatbot to acquire OSCE skills, making it an extremely important first step to put measures in place for a chatbot used in OSCE training. These measures must ensure that the chatbot is providing users with accurate and relevant information and is in line with the curriculum and program objectives for OSCE training. It is anticipated that the results can be improved since this study was conducted early in the chatbot deployment process. To ensure that the bot satisfies the requirements outlined in the paper’s first part, review, and discussion with users (students) are crucial. More clinical scenarios (with different frameworks) must be created to broaden the options available to students. The increase in sample size and understanding of differences in students’ performance regarding sex and curricular years is also a research question to be answered in the future. A module for instant feedback will also be implemented to maximise student learning and add voice communication to increase similarity to clinical settings. The integration of ChatGPT API to further improve the system is also one of the future projects.

## Data Availability

The datasets generated during and/or analyzed during the current study are not publicly available due confidentiality issues but are available from the corresponding author on reasonable request.

## Declarations

Ethics approval and consent to participate.

## Appendix

**Table A1.** Example of a history taking scheme focused on the symptom ‘leg pain’.

(Fishman & Fishman, 2010; Matthys et al., 2009; Peart, 2022)		
Initiating the Interview	Presentation	Ask for permission to talk with the patient after introducing yourself and identifying them (e.g., **name** and **age**). Ask the patient for their consent **prior** to star asking questions.
	Main complaint	To better understand the patient’s issue, pose open-ended questions regarding what brought them here, for example **leg pain**.
Gathering information	Explore the main complaint	Any kind of pain history can be described by the abbreviation **SOCRATES**. **S**ite: Where is the pain? **O**nset: When did it start? Was it constant/intermittent, gradual/sudden? **C**haracter: What is the pain like (sharp, burning, tight)? **R**adiation: Does it radiate anywhere? **A**ssociations: Is there anything else associated with the pain (for example, trauma? **T**ime course: How long did it last? **E**xacerbating: Does anything make it worse? **S**everity: How severe is the pain, using the 1–10 scale? **Avoid** asking multiple questions at once.
	Previous clinical history	Obtain details regarding a patient’s previous **health issues** or any previous **hospitalizations**. Screen for **MITJTHREADS**. **M**yocardial Infarction, **T**hromboembolism, **J**aundice, **T**uberculosis, **H**ypertension, **R**heumatic fever, **E**pilepsy, **A**sthma, **D**iabetes, **S**troke. Learn what **medications** the patient is taking, their dosage, and how frequently they take them. Find out whether the patient has any **allergies** and **vaccination** history.
	Social history	Inquire about **drinking** and **smoking**. Investigate the patient’s use of any **illicit substances** as well. Seek clarification about the patient’s **eating** and **exercise** routines, sexual history, employment, housing, marital status, sleeping patterns, overseas travelling and **stress** factors.
	Family history	Gather details regarding the patient’s family medical history, such as **diabetes, heart** history or **oncologic** conditions. Ask whether the family has any **hereditary** disorders.
Patients Perspective and planning	Interview closing	Review what the patient has told you repeating back the **key elements**, for the patient to correct you if there are any misunderstandings or mistakes. Address what the patient thinks is wrong with them and what they expect from the consultation. Use the **ICE** acronym: **I**deas, **C**oncerns and **E**xpectations. After the patient has finished asking any questions they may have, and you are satisfied with all the information you need, thank them for their time.

**Appendix 2- Example of a conversation between the student and the chatbot.**
